# Secondary Transmission of Coronavirus Disease from Presymptomatic Persons, China

**DOI:** 10.3201/eid2608.201142

**Published:** 2020-08

**Authors:** Weiwei Zhang, Weibin Cheng, Lei Luo, Yu Ma, Conghui Xu, Pengzhe Qin, Zhoubin Zhang

**Affiliations:** Guangzhou Center for Disease Control and Prevention, Guangzhou, China (W. Zhang, L. Luo, Y. Ma, C. Xu, P. Qin, Z. Zhang);; Guangdong Second Provincial General Hospital, Guangzhou (W. Cheng)

**Keywords:** respiratory infections, severe acute respiratory syndrome coronavirus 2, SARS-CoV-2, SARS, COVID-19, 2019 novel coronavirus disease, coronavirus disease, zoonoses, viruses, coronavirus secondary transmission, presymptomatic, secondary attack rate, China

## Abstract

We explored the secondary attack rate in different types of contact with persons presymptomatic for coronavirus disease (COVID-19). Close contacts who lived with or had frequent contact with an index case-patient had a higher risk for COVID-19. Our findings provide population-based evidence for transmission from persons with presymptomatic COVID-19 infections.

Coronavirus disease (COVID-19) caused by severe acute respiratory syndrome coronavirus 2 (SARS-CoV-2) is rapidly spreading across the globe. Some case reports and modeling studies suggest asymptomatic carriage of SARS-CoV-2 plays a role in transmission (*1*–*3*). Studies have shown that 30%–59% of SARS-CoV-2 infections are asymptomatic (*3*,*4*), which poses tremendous infection control challenges. To control asymptomatic infections, China implemented active case surveillance and enhanced social distancing measures, which include contact tracing, quarantine for key populations, medical observation, and curtailed social activities (*5*). However, additional information on the characteristics of presymptomatic transmission is needed to develop targeted control and prevention guidance.

We analyzed contact-tracing surveillance data collected during January 28–March 15, 2020, to explore the secondary attack rate from different types of contact with persons presymptomatic for COVID-19 in Guangzhou, China. Asymptomatic COVID-19 cases were found mainly through close contact screening, clustered epidemic investigations, follow-up investigation of infection sources, and active surveillance of key populations with travel or residence history in areas with continuous transmission of COVID-19 in China and abroad. We developed a case definition for presymptomatic COVID-19, criteria for close contact, and contact investigation and management guidelines (Appendix). We estimated secondary attack rate (SAR) and 95% CI based on the proportion of COVID-19 incidence among close contacts. We calculated the mean reproductive number (R_0_) from the number of secondary infections observed among close contacts of each index case. The study was approved by the ethics committee of Guangzhou Center for Disease Control and Prevention, which granted a waiver for informed consent. Data collection was conducted under the authority of the China Center for Disease Control and Prevention.

As of March 15, a total of 359 COVID-19 cases were confirmed in Guangzhou. Among them, 83 (23%) persons were asymptomatic at diagnosis; 71 (86%) of whom later developed symptoms. Among presymptomatic cases, 38 had >1 (range 1–90, median 4) close contact. We identified and included 369 close contacts in this study. Median age of close contacts was 35 years (range 0–93 years), 23.8% were family members of an index case, and 12 were confirmed to be infected via nucleic acid testing. Among them, 8 close contacts developed symptoms, and 4 were asymptomatic at the time of this study (Appendix Table). 

The overall SAR was 3.3% (95% CI 1.9%–5.6%). The SAR among household contacts was 16.1% and was 1.1% for social contacts, and 0 for workplace contacts. Older close contacts had the highest SAR compared with other age groups; 8.0% in persons >60 years of age compared with 1.4%–5.6% in persons <60 years of age. Close contacts of asymptomatic index case-patients had the lowest SAR, 0.8%, but the SAR was 3.5% for those with mild symptoms, 5.7% for those with moderate symptoms, and 4.5% for those with severe symptoms. Close contacts that lived with an index case-patient had 12 times the risk for infection and those who had frequent contact with an index case-patient, >5 contacts during 2 days before the index case was confirmed, had 29 times the risk for infection (Table).

**Table:** Characteristics of and secondary attack rates among 369 close contacts of persons with presymptomatic coronavirus disease 2019, China*

Variable	No. contacts	No. infected	Attack rate, % (95% CI)	Relative risk (95% CI)
Sex				
M	217	5	2.3 (0.1–5.2)	Referent
F	152	7	4.6 (2.2–8.9)	2.1 (0.6–6.6)
Age				
≤17	46	2	4.3 (1.2–14.5)	Referent
18–30	104	3	2.9 (1.0–8.1)	0.7 (0.1–4.1)
31–40	72	1	1.4 (0.2–7.4)	0.4 (0.03–3.5)
41–50	68	1	1.5 (0.3–7.9)	0.4 (0.03–3.7)
51–60	54	3	5.6 (1.9–15.1)	1.3 (0.2–8.1)
≥61	25	2	8.0 (1.4–27.5)	1.9 (0.3–14.5)
Index case-patient status*				
Asymptomatic	119	1	0.8 (0.2–5.6)	Referent
Mild symptoms	141	5	3.5 (1.5–8.0)	4.3 (0.5–37.7)
Moderate symptoms	87	5	5.7 (2.5–12.8)	7.2 (0.8–62.7)
Severe symptoms	22	1	4.5 (0.8–21.8)	5.6 (0.3–93.4)
Contact mode				
Social interaction with friends or relatives	66	1	1.5 (0.3–8.1)	Referent
Lived together	62	10	16.1 (9.0–27.2)	12.5 (1.6–100.8)
Worked together	119	0	0	0
Social interaction with strangers	122	1	0.8 (0.2–4.9)	0.5 (0.03–8.7)
Contact frequency†				
Rare	149	1	0.7 (0.1–3.7)	Referent
Moderate	159	1	0.6 (0.1–3.5)	0.9 (0.1–15.1)
Frequent	61	10	16.4 (9.2–27.6)	29.0 (3.6–232.3)
*Status as of March 30, 2020, based on the person’s clinical course assessed by a physician. Moderate symptoms included fever, respiratory symptoms, and radiographic evidence of pneumonia. Severe symptoms included breathing rate >30/min; oxygen saturation level <93% at rest; oxygen concentration level PaO_2_/FiO_2_ <300 mmHg (1 mmHg = 0.133kPa); lung infiltrates >50% within the past 24–48 h; respiratory failure requiring mechanical ventilation; septic shock; or multiple organ dysfunction or failure. All other symptomatic cases were classified as mild. †Rare contact was defined as contacted with index cases <2 times during 2 days preceding confirmation of infection. Moderate contact was defined as contacted with index cases 3–5 times during 2 days preceding confirmation of infection. Frequent contact was defined as contacted with index cases >5 times during 2 days preceding confirmation of infection.				

Our findings substantiate previous reports from China and Germany (*1*,*2*,*6*) and show that SARS-CoV-2 can be transmitted during asymptomatic COVID-19 infection period. The probability of infection increased substantially among close contacts who shared living environments or had frequent contact with an index case-patient, which underlines the need for prompt contact-based surveillance and social distancing (*7*). Our results also showed most secondary infections occurred in confined familial clusters and that persons >60 years of age appear to be more vulnerable to being infected. These results are consistent with previous reports on epidemiologic characteristics of 72,314 COVID-19 cases in China (*8*) and suggest that household-based isolation should be cautiously implemented for persons with asymptomatic suspected cases. We also noted that persons with asymptomatic infections appeared to be less effective in transmitting the virus. However, this finding should not discourage isolation and surveillance efforts. The R_0_ in this cohort was 0.3 (95% CI 0.2–0.5), which was far smaller than the overall R_0_ of 2.2 reported previously (*9*). This low transmission level could be the result of active surveillance, centralized quarantine, and forceful social-distancing strategies in Guangzhou.

Interpretation of the findings should be taken with caution, and several limitations influence our estimation of the SAR. First, the number of close contacts was limited because we only included those who had been reached, and asymptomatic infections might have been missed. Second, we excluded close contacts who were exposed to >2 confirmed COVID-19 case-patients. Third, the presymptomatic transmission period is not well defined. 

Despite these limitations, our analysis provides valuable information on secondary transmission of SARS-CoV-2 in different types of contact with presymptomatic COVID-19 case-patients. Further evidence is needed to define the population characteristics, communicable period, and the volume and duration of viral shedding from persons with asymptomatic infections.

Appendix Additional information on secondary transmission of coronavirus disease from presymptomatic persons, China
