# Supplementary material for: Secondary Transmission of Coronavirus Disease from Presymptomatic Persons, China
Source: Emerg Infect Dis. 2020 Aug;26(8):1924–6. doi: 10.3201/eid2608.201142 (PMC7392433; doi:10.3201/eid2608.201142)
Supplement: Appendix — Additional information on secondary transmission of coronavirus disease from presymptomatic persons, China [file 20-1142-Techapp-s1.pdf]

Article DOI: https://doi.org/10.3201/eid2608.201142

Secondary Transmission of Coronavirus Disease from Presymptomatic Persons, China

Appendix

Presymptomatic Coronavirus Disease (COVID-19) Case Definition

We defined presymptomatic COVID-19 cases (index case) as the persons with positive nucleic acid tests for severe acute respiratory syndrome coronavirus 2 (SARS-CoV-2), the causative agent of COVID-19, but without relevant clinical symptoms, such as fever, cough, sore throat, and other symptoms and signs that persons could self-perceive or that could be clinically identifiable. We included 2 types of presymptomatic COVID-19 cases. For one, the asymptomatic person has a positive nucleic acid test, but after the 14-day incubation period the person remains asymptomatic; no self-perceived or clinically recognized symptoms or signs ever manifest. For the other, the asymptomatic person has a positive nucleic acid test, and has no self-perceived or clinically recognizable symptoms and signs at the time of sampling, but the person later develops clinical manifestations during medical observation, i.e., a latent period of "asymptomatic infection." Asymptomatic COVID-19 cases were found mainly through screening of close contacts, clustered epidemic investigations, follow-up investigation of infection sources, and active surveillance of key populations with a history of travel to or residence in areas with continuous transmission of COVID-19. We only included secondary cases that met the presymptomatic COVID-19 case criteria because they could still have a third round of transmission, but this did not happen in our cases.

Close Contact Criteria

According to China Center for Disease Control (China CDC) guidelines for COVID-19 close contact investigation and management, close contact of asymptomatic COVID-19 case refers to a person who had contact with index case without using proper protection during 2 days before the index case was tested. Proper protection usually refers to wearing a surgical mask consistently while in contact with the index case. Contact investigation interviews were conducted by trained Guangzhou CDC epidemiologists to collect information on the number of close contacts, the mode and frequency of contact, and other factors. Close contacts include any of the following: family members living together in the same household; direct caregivers or those providing medical treatment and nursing services; medical staff carrying out care activities that might generate aerosols in the same space; people sharing a small place, such as an office, workshop, elevator, cafeteria, or classroom; persons sharing meals or visiting together, or those providing catering and entertainment services in a closed environment; medical personnel, family members, or other persons who have close contact with the case; sharing transportation, such as in vehicle, or on a flight or ship, and having close contact with an index case, which includes passengers, attendants, or companions, such as family members, colleagues, or friends.

After interviews, the on-site investigators assessed whether additional persons might meet the criteria for close contact. We excluded close contacts when there was shared exposure to $\geq$2 confirmed COVID-19 cases.

Contact Management

Close contacts were put in quarantine at a centralized facility, such as hotel, housed in separate rooms, and underwent medical observation for 14 days. All close contacts had nucleic acid testing at the beginning and end of the medical observation. Extra testing was provided for those who developed symptoms during quarantine.

Management Process

1. Informed consent. When carrying out medical observation, provide written or oral information to the contact on rationale, duration, legal basis, precautions, and disease-related reason for medical observation, and the medical and health institutions, contact persons, and contact information of entities responsible for medical observation.

2. Health monitoring. Each morning and evening, the staff of the designated medical and health institutions take temperature measurements of the close contacts, ask about their health status, and give necessary help and guidance.

3. Observation period. The medical observation period is 14 days after the last effective contact with the case without taking proper protection. If a close contact tests negative during medical observation, he or she still needs to continue observation until the 14-day period expires.

4. Treatment for abnormal symptoms. During medical observation, the close contact should immediately report any symptoms, such as fever, dry cough, or other respiratory symptoms; diarrhea; or conjunctival congestion, to the local health department. Then the contact will be sent to the designated medical institution for treatment and further testing. Additional close contacts of suspected, clinically diagnosed, and confirmed cases, will be traced and put in quarantine.

5. Lift medical observation. When the medical observation period expires, close contacts who remain asymptomatic will be released if they have 2 negative nucleic acid tests conducted >24 hours apart. Close contacts can be released prior to the 14-day observation period if the suspected index case is ruled out.

Appendix Table. Characteristics of 12 secondary cases of coronavirus disease (COVID-19) transmitted by asymptomatic persons, China

Index cases*		Secondary cases					
Sex	Symptoms	Age, y/ sex	Relationship to index case	Contact mode	Symptoms	Incubation period, d†	Serial interval‡
M	Mild	23/M	Brother-in-law	Lived together	Moderate	8	11
M	Mild	24/F	Girl friend	Lived together	Asymptomatic	–	–
M	Mild	3/M	Son	Lived together	Mild	6	1
F	Moderate	58/F	Friend	Social interaction	Moderate	6	8
M	Mild	54/F	Mother-in-law	Lived together	Mild	9	12
M	Severe	79/F	Wife	Lived together	Mild	7	7
F	Moderate	57/M	Husband	Lived together	Moderate	11	9
F	Mild	44/F	Mother	Lived together	Mild	6	1
F	Moderate	63/F	Mother-in-law	Lived together	Moderate	10	9
F	Moderate	36/F	Wife	Lived together	Asymptomatic	–	–
F	Moderate	3/M	Son	Lived together	Asymptomatic	–	–
M	Asymptomatic	30/M	Colleague	Social interaction	Asymptomatic	–	–

*Index cases were asymptomatic at time of contact. Symptom severity of index cases are reported at time of COVID-19 diagnosis.

†Incubation period was defined as the interval between last contact with asymptomatic persons later identified as COVID-19 index cases and the onset of clinical signs and symptoms. Incubation period was estimated only for cases that had onset of signs and symptoms.

‡Serial interval was defined as the interval between onset of signs and symptoms in index case and secondary case. We calculated serial intervals only for pairs in which the index and secondary cases both had clinical manifestations
